# Characterization of Metabolic Patterns in Mouse Spermatogenesis and Its Clinical Implications in Humans

**DOI:** 10.3390/ijms26031001

**Published:** 2025-01-24

**Authors:** Jiachen Wang, Mengqi Chen, Ying Yao, Mengyuan Zhu, Yingtong Jiang, Jiawei Duan, Yan Yuan, Laihua Li, Minjian Chen, Jiahao Sha

**Affiliations:** 1 State Key Laboratory of Reproductive Medicine and Offspring Health, Nanjing Medical University, Nanjing 211166, China; wjcchonya@163.com (J.W.); chenmq_njmu@outlook.com (M.C.); yaoyimg@163.com (Y.Y.); yuanyan@njmu.edu.cn (Y.Y.); laihuali2023@njmu.edu.cn (L.L.); 2 Key Laboratory of Modern Toxicology of Ministry of Education, School of Public Health, Nanjing Medical University, Nanjing 211166, China; tanshuizhu123@outlook.com (M.Z.); ytjiang61@163.com (Y.J.); jwduan1997@163.com (J.D.)

**Keywords:** metabolomics, spermatogenesis, Leydig cells, Sertoli cells, azoospermia, seminal plasma

## Abstract

Spermatogenesis is a complex process requiring precisely controlled metabolic adaptations. Although the genetic and cellular aspects of spermatogenesis have been extensively studied, the underlying metabolic mechanisms remain largely underexplored. In this study, we utilized STA-PUT technology to separate three key cell types involved in mouse spermatogenesis: pachytene spermatocytes (PAC), round spermatids (RS), and elongated spermatids (ES). A comprehensive untargeted metabolomic analysis revealed significant metabolic changes during spermatogenesis, such as reduced methylation-related metabolites and increased glycolytic intermediates and TCA cycle metabolites during ES. Moreover, metabolic differences between germ cells and somatic cells (Leydig and Sertoli cells) were highlighted, particularly in steroidogenesis and lipid metabolism. To investigate clinical relevance, we analyzed human seminal plasma. Samples from individuals with azoospermia displayed significant metabolic abnormalities, including reduced methionine, tryptophan, and arginine, which play vital roles in sperm development. Pathway enrichment analysis revealed disturbances in the metabolism of nucleotide, amino acid, and energy in azoospermia, suggesting potential biomarkers of male infertility. Our findings provide a comprehensive metabolic profile of spermatogenesis and suggest that metabolic alterations may be significant contributors to male infertility, particularly in cases of azoospermia.

## 1. Introduction

Spermatogenesis is an intricate and multistage process in which spermatogonia stem cells divide to the round spermatids and transform into mature sperm, and is crucial for male fertility and reproductive health. This multifaceted progression encompasses precise transitions through various cell types, including pachytene spermatocytes (PAC), round spermatids (RS), and elongated spermatids (ES), each exhibiting unique structural and functional properties [1]. Despite advances in comprehending the genetic and cellular aspects of spermatogenesis, the metabolic landscape across various stages remains inadequately explored. This leaves a significant gap in the knowledge regarding how specific metabolic pathways contribute to cellular differentiation during this process.

Metabolomics is a discipline focused on the study of small molecular metabolites, such as carbohydrates, lipids, amino acids, and nucleotides, within biological systems. Through quantitative analysis of these metabolites, metabolomics provides insights into metabolic alterations that occur in various physiological and pathological conditions. By employing high-throughput screening techniques on biological samples such as blood, urine, and tissues, metabolomics helps identify metabolic alterations and potential biomarkers [2]. Furthermore, it also offers novel diagnostic and therapeutic tools in clinical medicine, facilitating early disease diagnosis, prognostic assessment, and treatment monitoring [3]. The production of fresh cellular metabolites are key regulators of germ cell fate and functions, not merely byproducts of cellular activity. In spermatogenesis, mitochondria regulate stem cell fate decisions and lineage determination. This occurs through various mechanisms, including signal transduction, protein modifications, and epigenetic modulations [4]. Research suggests that the pentose phosphate pathway and Ser-Gly-one-carbon metabolism may contribute to germ cell properties at various developmental stages [5]. Previous studies have identified changes in essential metabolites and pathways, including glycolysis, oxidative phosphorylation, and lipid metabolism, during the initial phases of spermatogonial differentiation and later stages of sperm maturation [6,7]. Additionally, metabolomic studies across various species have provided insights into metabolic regulation and potential disruptions in reproductive diseases [8,9]. However, these studies have emphasized singular pathways or a limited range of cell types, leading to a partial and less comprehensive view of the metabolic changes that support spermatogenesis at each stage of cellular development. Therefore, integrating metabolomic analysis into the study of cellular markers could reveal metabolic requirements specific to each cell type and provide valuable insights into the cellular mechanism underlying successful spermatogenesis.

This study utilized a metabolomics approach to investigate metabolic progression across different spermatogenic stages, including PAC, RS, and ES, along with testicular somatic cells, such as Sertoli and Leydig cells, to address the critical knowledge gaps in our understanding of the metabolic pathways underlying spermatogenesis. We isolated these cellular groups from mouse testes and conducted metabolic profiling using ultra-high-performance liquid chromatography with mass spectrometry (UPLC-MS). Further, orthogonal partial least squares discriminant analysis (OPLS-DA) was applied to visualize metabolic transitions and determine stage-specific metabolites.

The analysis revealed metabolic adaptations that facilitate cellular changes, emphasizing key pathways such as glycolysis, the tricarboxylic acid cycle, and arginine metabolism, which meet the energy requirements of spermatogenesis. This integrative approach offers a comprehensive view of the metabolic landscape in mouse spermatogenesis. Moreover, we extended our research to include human seminal plasma, comparing samples from normal male controls and patients with azoospermia to explore clinical relevance. The azoospermia samples showed significant disruptions in nucleotide, amino acid, and energy metabolisms, suggesting potential biomarkers for male infertility.

## 2. Results

### 2.1. Metabolic Dynamics and Cell Type Identification in Mouse Spermatogenesis

**Figure 1:**
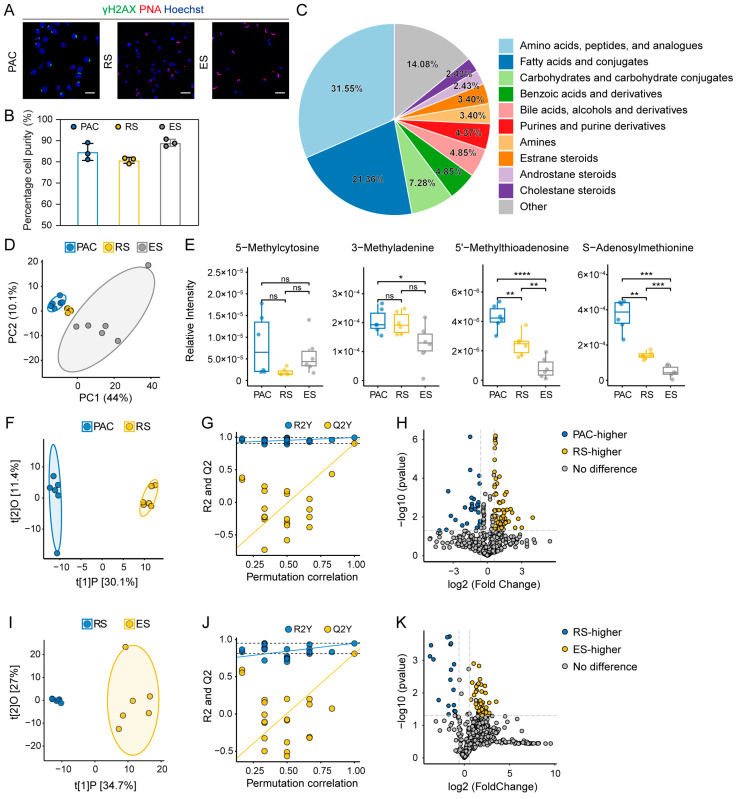
Metabolic shifts from the early stages of spermatogenesis to maturation of the sperm of mouse. (**A**) Immunofluorescence staining for γH2AX (green) and PNA (red) in different spermatogenic cell types. Nuclei are stained with Hoechst 33342 (blue). Scale bars, 20 μm. PAC, pachytene spermatocytes; RS, round spermatids; ES, elongated spermatids. (**B**) Histogram showing the purity of PAC, RS, and ES isolated using the STA-PUT method. Data represented as mean ± SD. (**C**) Pie chart showing the classification of the metabolites identified, focusing on categories containing more than three metabolites. (**D**) Principal component analysis (PCA) of the metabolomic profiles of germ cells. (**E**) Box plots showing the relative intensities of key methylation-related metabolites, including 5-methylcytosine, 3-methyladenine, 5′-methylthioadenosine, and S-adenosylmethionine, across PAC, RS, and ES stages. Statistical significance was determined using an independent *t*-test. The boxes represent the interquartile range (IQR), with the horizontal line indicating the median value. Whiskers extend to 1.5 times the IQR, and individual data points are shown as jittered dots. ns, *p*-value ≥ 0.05; *, *p*-value < 0.05; **, *p*-value < 0.01; ***, *p*-value < 0.001; ****, *p*-value < 0.0001, respectively. (**F**) OPLS-DA score plots showing clear separations between PAC and RS (R^2^X = 0.415, R^2^Y = 0.994, Q^2^ = 0.903). (**G**) OPLS-DA model in (**F**) validation with permutation test, showing R2Y and Q2Y values with dashed lines indicating their maximum values. (**H**) Volcano plot comparing differential metabolites between PAC and RS. Metabolites enriched in PAC (blue) and RS (yellow) are highlighted. Dashed lines represent thresholds of log2 fold change and *p*-value. (**I**) OPLS-DA score plots showing clear separations between RS and ES (R^2^X = 0.617, R^2^Y = 0.948, Q^2^ = 0.81). (**J**) OPLS-DA model in (**I**) validation with permutation test, showing R2Y and Q2Y values with dashed lines indicating their maximum values. (**K**) Volcano plot comparing differential metabolites between RS and ES. Metabolites enriched in RS (blue) and ES (yellow) are highlighted. Dashed lines represent thresholds of log2 fold change and *p*-value.

To characterize metabolic changes occurring during spermatogenesis, we employed the STA-PUT method to isolate three key cell types (PAC, RS, and ES) involved in mouse spermatogenesis [10]. Furthermore, to validate the cell type identification and purity, we employed immunofluorescence staining analyses. The results demonstrate that PAC showed a positive expression of γH2AX [11], a marker related to meiotic sex chromosome regulation, while both RS and ES displayed positive results for peanut agglutinin (PNA) [10], an established acrosome marker (Figure 1A). The average purity of PAC, RS, and ES isolated through the STA-PUT method exceeded 80% (Figure 1B), further ensuring the reliability of downstream analyses.

Subsequently, we performed a metabolomic analysis of spermatogenesis by utilizing UPLC-MS. The identified metabolites were predominantly classified into several major categories: amino acids and their analogs, fatty acids and their conjugates, and carbohydrates and their conjugates (Figure 1C). Principal Component Analysis (PCA) revealed distinct metabolomic profiles for PAC, RS, and ES, with PC1 (44%) and PC2 (10.1%) representing the majority of the variance in the data (Figure 1D). These findings collectively indicate our successful establishment of a metabolic landscape for PAC, RS, and ES during spermatogenesis in mice.

### 2.2. Metabolomic Alterations During Spermatogenesis

The dynamic regulation of DNA methylation influences gene expression and developmental processes throughout various stages of spermatogenesis [12]. We studied the sequential key methylation-related metabolic changes that occur during the different stages of spermatogenesis. The results reveal that key methylation-related metabolites, such as 3-methyladenine, S-adenosylmethionine, and 5′-methylthioadenosine, exhibited significant decreases across different stages, particularly in ES, which is consistent with the previous findings [13]. Although 5-methylcytosine did not display significant differences between stages, its concentration was noticeably reduced in ES (Figure 1E).

Next, we investigated the sequential metabolic alterations that occur at different stages of spermatogenesis. OPLS-DA analysis revealed clear metabolic separations between PAC and RS groups, as well as between RS and ES groups (Figure 1F,I). The results highlight the apparent metabolic differences among the different cell types and a high degree of internal homogeneity within each individual cell type. The stability and predictive power of the OPLS-DA model were confirmed by permutation testing, showing its effectiveness in identifying the metabolic characteristics among different cell types (Figure 1G,J).

A substantial number of metabolites exhibited significant upregulation or downregulation in their levels using the selection criteria of fold change >1.5, Variable Importance in Projection (VIP) > 1, and *p* < 0.05. The results reveal that 26 metabolites with higher concentrations in PAC, while 70 metabolites were more abundant in RS, indicating significant metabolic changes occurring during the PAC to RS transition (Figure 1H, Supplementary Table S1). Meanwhile, during the process of sperm maturation, 17 metabolites showed higher concentrations in RS, while 50 were higher in ES (Figure 1K, Supplementary Table S2).

The findings emphasize the distinct metabolic reprogramming during the PAC to RS transition and the maturation of sperm.

### 2.3. Key Metabolic Pathways During Spermatogenesis

**Figure 2:**
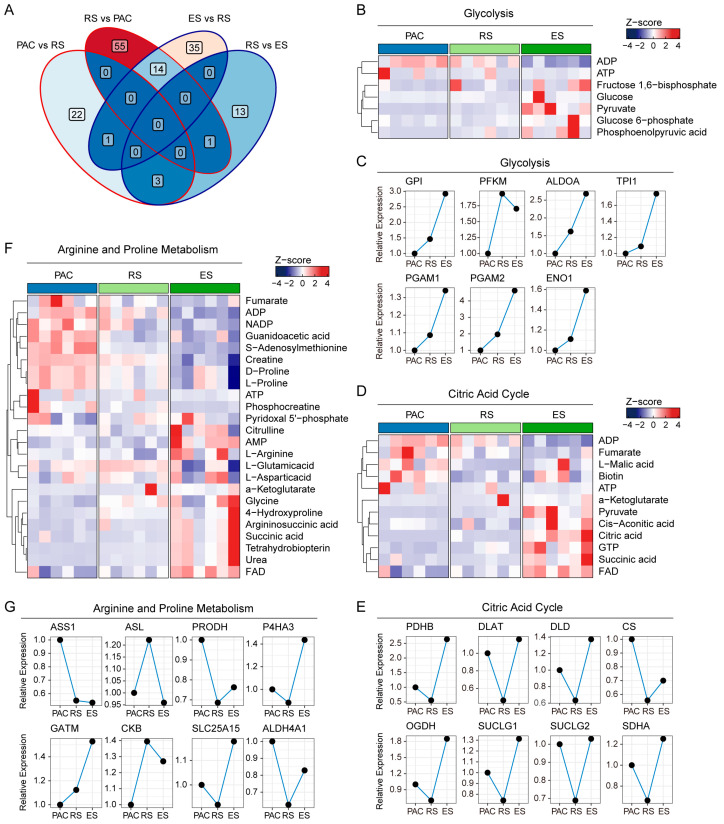
Key metabolic pathways during spermatogenesis of mouse. (**A**) Venn diagram showing the overlap of differentially up-regulated metabolites during spermatogenesis. (**B**,**C**) Heatmap and line plots showing the changes in metabolites (**B**) and enzymes (**C**) involved in glycolysis across the stages of spermatogenesis. (**D**,**E**) Heatmap and line plots showing the dynamic changes in metabolites (**D**) and enzymes (**E**) involved in the citric acid cycle across the stages of spermatogenesis. (**F**,**G**) Heatmap and line plots showing the dynamic changes in metabolites (**F**) and enzymes (**G**) involved in arginine and proline metabolism across the stages of spermatogenesis.

We performed a Venn analysis to identify the overlap of differential metabolites between germ cells at different stages of mouse spermatogenesis. We found that PAC and RS, as well as ES and RS, share 14 differential metabolites (Figure 2A). Based on this observation, we conducted a cross-stage analysis of the key metabolic pathways involved in spermatogenesis. To gain a comprehensive understanding of the changes in the key metabolic pathways, we utilized the proteomic data from previous studies on spermatogenesis [14]. The results show that, during the ES stage, there is a significant elevation in the levels of key glycolytic intermediates, including fructose-1,6-bisphosphate, glucose, glucose-6-phosphate, and phosphoenolpyruvic acid, indicating a strong activation of the glycolysis process in sperm maturation (Figure 2B). The ES stage is characterized by the accumulation of these metabolites and upregulation in the expression of core glycolytic enzymes, including GPI (Glucose-6-Phosphate Isomerase), PFKM (Phosphofructokinase), ALDOA (Aldolase, Fructose-bisphosphate A), TPI1 (Triosephosphate Isomerase 1), PGAM1/2 (Phosphoglycerate Mutase 1/2), and ENO1 (Enolase 1). The presence of these enzymes at this developmental stage evidenced the activation of multiple critical steps of the glycolytic pathway (Figure 2C), while the ES stage is characterized by the upregulation of the expression of components of the pyruvate dehydrogenase complex, including PDHB (Pyruvate Dehydrogenase E1 Subunit Beta), DLAT (Dihydrolipoamide S-Acetyltransferase), and DLD (Dihydrolipoamide Dehydrogenase) (Figure 2E). This indicates an increased pyruvate metabolism and its mitochondrial conversion to acetyl-CoA, which subsequently serve as fuels for the tricarboxylic acid cycle (TCA cycle/citric acid cycle) (Figure 2D).

Similarly, a significant increase was observed in key metabolites associated with the TCA cycle, such as pyruvate, cis-aconitic acid, citric acid, succinic acid, and FAD (flavin adenine dinucleotide), which provide additional support to the enhanced activity of this pathway. In line with these metabolic changes, the expression levels of key TCA cycle enzymes also elevated during the ES stage, including OGDH (Oxoglutarate Dehydrogenase), SDHA (Succinate Dehydrogenase Complex Flavoprotein Subunit A), SUCLG1 (Succinate-CoA Ligase GDP/ADP-Forming Subunit Alpha), and SUCLG2 (Succinate-CoA Ligase ADP-Forming Subunit Beta). The RS stage, predominantly characterized by the completion of meiosis, exhibits lower energy requirements than the proliferative PAC stage and the metabolically active ES stage. As a result, the expression levels of TCA cycle-related enzymes are significantly reduced during the RS stage compared to the PAC and ES stages (Figure 2E).

Arginine is a key molecule in the nitrogen metabolism and a precursor for nitric oxide (NO) synthesis, both of which play vital physiological roles during the process of spermatogenesis [15,16]. The stage-specific high expression of ASS1 (Argininosuccinate Synthase 1) and ASL (Argininosuccinate Lyase) contributes to the increased levels of L-arginine observed in the ES stage (Figure 2F,G). The concurrent increase in tetrahydrobiopterin (BH_4_), a substrate for NO synthesis and an essential cofactor for NO synthase, provides additional evidence for the active synthesis of NO, which contributes to the functional maturation of sperm. The increased levels of urea, which is the end product of the arginine metabolism, indicate the enhanced activity of arginine metabolism during the ES stage (Figure 2F).

During the PAC stage, when cells primarily engage in cell division and differentiation, there is an immediate and concentrated demand for energy. Correspondingly, the levels of CKB (Creatine Kinase B), creatine, and phosphocreatine are elevated, allowing for the rapid regeneration of ATP to support cellular activities. In contrast, during the ES stage, the metabolic focus shifts toward the accumulation of arginine and glycine. Through the action of GATM (Glycine Amidinotransferase), these amino acids are converted into guanidinoacetic acid, thereby supplying the essential substrates for the subsequent creatine metabolic cycle.

In addition, the PAC stage is characterized by the synthesis and metabolism of D/L-proline (Figure 2F). The elevated expression of PRODH (Proline Dehydrogenase 1) and ALDH4A1 (Aldehyde Dehydrogenase 4 Family Member A1) supports the metabolic requirements for proline (Figure 2F,G). In the ES stage, besides the enzymes directly involved in arginine metabolism, other enzymes exhibit increased expression. P4HA3 (Prolyl 4-Hydroxylase Subunit Alpha 3) facilitates the hydroxylation of proline to 4-hydroxyproline (Figure 2G).

**Figure 3:**
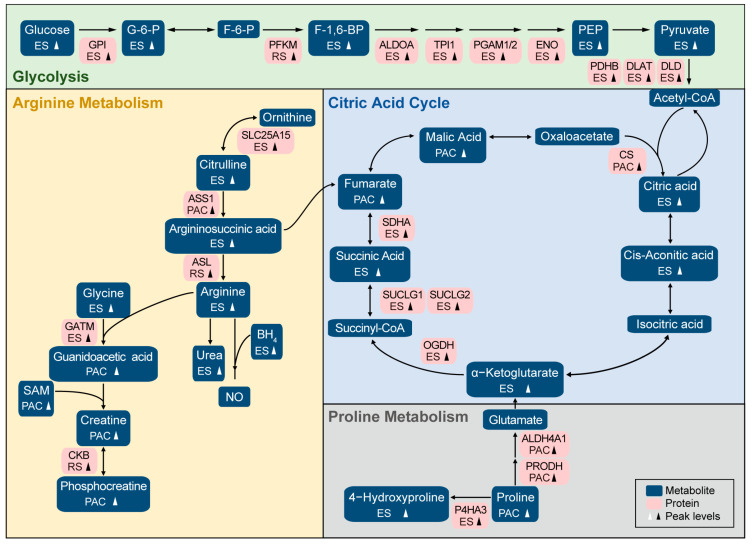
Activities of metabolic pathways during mouse sperm maturation. Schema diagram of metabolism during mouse sperm maturation, showing the key enzymes and their corresponding metabolites. Black and white arrows indicate peak levels at indicated stage. Blue boxes represent metabolites, and pink boxes represent the protein of indicated metabolic enzymes. G-6-P, Glucose-6−phosphate. F-6-P, Fructose-6−phosphate. F-1,6-BP, Fructose 1,6−bisphosphate. PEP, Phosphoenolpyruvic acid. SAM, S−Adenosylmethionine. BH_4_, Tetrahydrobiopterin. NO, Nitric oxide.

To understand the involvement of metabolic pathways in mouse spermatogenesis, we systematically mapped all enzymes, the key mediators of cellular metabolism, onto the principal metabolic pathways associated with their corresponding metabolites (Figure 3). These enzymes and their corresponding metabolites highlighted the consistency between metabolomic and proteomic findings, providing a comprehensive insight into metabolic regulation during sperm maturation.

### 2.4. Metabolomic Characteristics of Sertoli Cells and Leydig Cells

**Figure 4:**
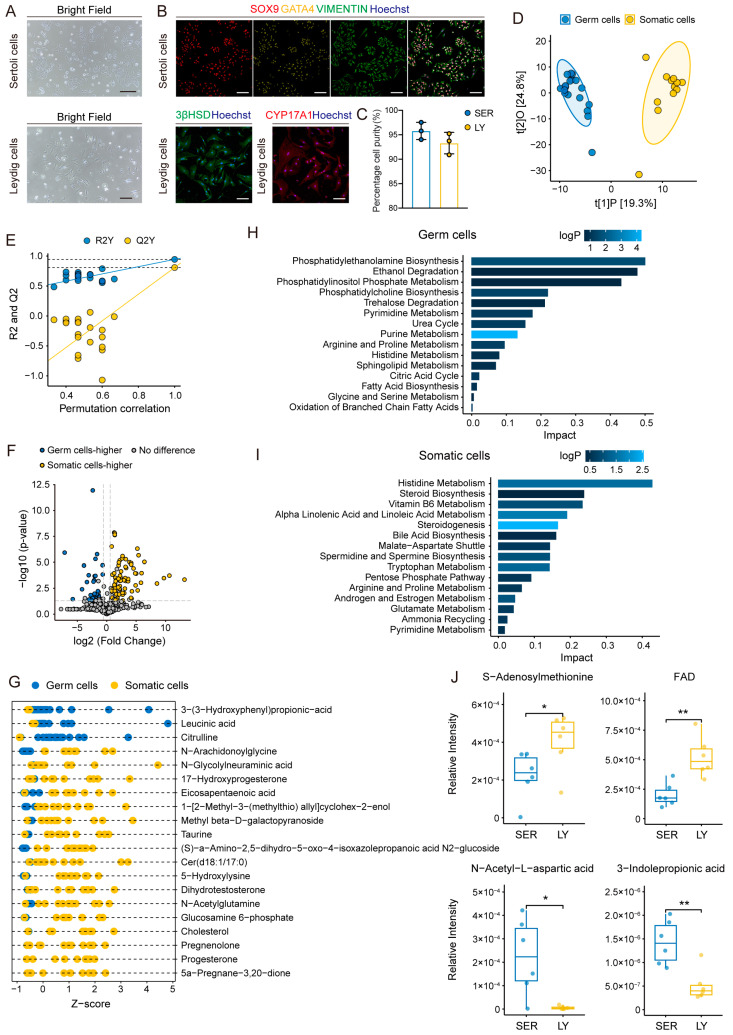
Comparative metabolomic profiling of mouse germ cells and somatic cells. (**A**) Bright-field microscopy images showing the distinct morphology of adherent cultures of Leydig cells and Sertoli cells. Scale bars, 200 μm. (**B**) Immunofluorescence staining confirming the identity of Leydig and Sertoli cells. Leydig cells are stained with 3βHSD (lime green), CYP17A1 (dark red). Sertoli cells are stained with VIMENTIN (green), GATA4 (yellow), SOX9 (red). Nuclei are stained with Hoechst 33342 (blue). Scale bars, 100 μm. (**C**) Histogram showing the purity of Sertoli cells and Leydig cells. Data represented as mean ± SD. (**D**) OPLS-DA score plot showing the separation between germ cells and somatic cells based on their metabolic profiles (R^2^X = 0.441, R^2^Y = 0.944, Q^2^ = 0.808). (**E**) Permutation validation plot of the OPLS-DA model in (**D**), showing R2Y and Q2Y values with dashed lines indicating their maximum values. (**F**) Volcano plot displaying differential metabolites between germ cells and somatic cells. Germ cell-enriched metabolites are highlighted in blue, while metabolites elevated in somatic cells are shown in yellow. Dashed lines represent thresholds of log2 fold change and *p*-value. (**G**) Z-score plot of the top 20 differential metabolites between germ cells and somatic cells. (**H**) Pathway impact analysis based on somatic cell-enriched metabolites. (**I**) Pathway impact analysis based on germ cell-enriched metabolites. (**J**) Box plots illustrating the relative intensities of selected metabolites that differ between Sertoli cells (SER) and Leydig cells (LY). Statistical significance was determined using an independent *t*-test. The boxes represent the IQR, with the horizontal line indicating the median value. Whiskers extend to 1.5 times the IQR, and individual data points are shown as jittered dots. *, *p*-value < 0.05; **, *p*-value < 0.01.

Sertoli and Leydig cells establish a complex hormonal and paracrine network system that regulates the entire spermatogenesis process from the spermatogonia stage to the mature sperm stage. Sertoli cells provide structural and nutritional support for the development of germ cells, establishing the blood–testis barrier and regulating germ cell development through paracrine signaling [17,18]. In contrast, Leydig cells produce testosterone and paracrine factors essential for germ cell progression and the proper functioning of Sertoli cells [19,20]. Additionally, we extracted two types of somatic cells (Sertoli cells and Leydig cells) from mouse testes (Figure 4A). Furthermore, Leydig cells were identified through immunofluorescence staining using 3βHSD^+^ and CYP17A1^+^ markers, while Sertoli cells were verified using VIMENTIN^+^, GATA4^+^, and SOX9^+^ markers. These identifications were based on the cells’ respective steroidogenic and supportive functions in spermatogenesis [21,22,23] (Figure 4B). Using immunofluorescence staining of these key markers, we confirmed that the purity of the collected Sertoli and Leydig cells exceeded 90%, further ensuring the reliability of downstream metabolic and functional analyses (Figure 4C).

To examine the metabolic differences between germ cells and somatic cells, we employed an OPLS-DA score plot. The results reveal a clear separation in the metabolic profiles of these two cell types, suggesting substantial differences in their metabolic characteristics (Figure 4D). The permutation test validated the effectiveness of the OPLS-DA model in differentiating the metabolic profiles among various cell types (Figure 4E). Analysis of the volcano plot reveals that somatic cells exhibit higher levels of 81 metabolites, whereas germ cells show increased concentrations of 28 metabolites (Figure 4F).

The high abundance of metabolites in germ cells is primarily concentrated in pathways associated with DNA synthesis, energy metabolism, and cell membrane formation. Our database (Supplementary Tables S3–S5) shows that certain metabolites, such as leucinic acid and citrulline, are found in significantly elevated levels within germ cells (Figure 4G). These identified metabolites are closely related to several metabolic pathways, including purine and pyrimidine metabolism, the urea cycle, TCA cycle, and phosphatidylethanolamine biosynthesis (Figure 4H). The heightened activity of purine and pyrimidine metabolism may be associated with the increased need for nucleotides during the rapid cell proliferation and DNA synthesis that occurs in germ cells. Furthermore, the increased activity of both the urea and TCA cycle indicates the substantial requirements needed for energy and amino acid synthesis during germ cell differentiation. This supports the rapid cell division and metabolic processes associated with spermatogenesis. Moreover, the significant upregulation of phosphatidylethanolamine synthesis suggests an increased need for the synthesis of membrane structures during sperm maturation [24].

In comparison, somatic cells display notably distinct metabolic characteristics related to steroid synthesis and lipid metabolism. The metabolic profile of these cells is characterized by elevated levels of steroid hormone precursors, such as 17-hydroxyprogesterone and pregnenolone (Figure 4G). These precursors are primarily enriched in pathways linked to steroidogenesis and linoleic acid metabolism (Figure 4I). The significant enrichment of steroidogenesis highlights the essential function of Leydig cells in testosterone production. These cells are important for the production of steroid hormones, especially testosterone, which is crucial for maintaining reproductive function and male sexual characteristics. On the contrary, Sertoli cells are likely more significant in regulating lipid metabolism, assisting in the formation of sperm cell membranes, and providing nutrients during spermatogenesis.

Subsequently, we analyzed the metabolic differences between Sertoli and Leydig cells, uncovering unique metabolic profiles (Figure 4J). S-adenosylmethionine, a primary methyl donor, participates in various biochemical reactions in the body. FAD, an important coenzyme in many redox reactions, is crucial in the mitochondrial electron transport chain and metabolic energy processes. The increased levels of these metabolites align with the role of Leydig cells in producing hormones such as testosterone, a process that requires substantial energy and precise regulation through methylation-based modifications [25]. Conversely, N-acetyl-L-aspartate (NAA) is generally considered a metabolite associated with energy storage and signaling in the nervous system [26], and may function in Sertoli cells. These cells, also known as “nurse cells,” are important in providing metabolic and energy support to germ cells throughout the process of spermatogenesis. Additionally, 3-indolepropionic acid (IPA), an antioxidant metabolite, is enriched in Sertoli cells, potentially indicating the cells’ function in protecting germ cells from oxidative stress [27,28]. The findings reveal significant metabolic differences between germ and somatic cells, suggesting a synergistic relationship between these cell types during spermatogenesis.

### 2.5. Metabolomic Profiling of Human Seminal Plasma Reveals Key Metabolic Alterations in Azoospermia

**Figure 5:**
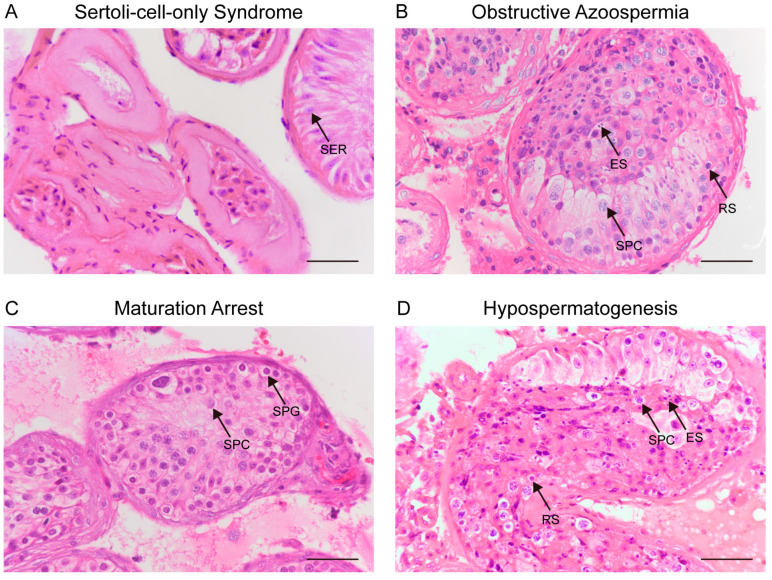
H&E staining images of testicular biopsy tissue. (**A**–**D**) Representative testicular tissue from (**A**) Sertoli cell only (SCO) syndrome patients, (**B**) obstructive azoospermia (OA) patients, (**C**) sperm maturation arrest (MA) patients, and (**D**) hypospermatogenesis (HS) patients. SER, Sertoli cell; SPG, spermatogonia; SPC, spermatocytes; RS, round spermatids; ES, elongated spermatids. Scale bar, 50 μm.

To validate the clinical relevance of metabolic changes during spermatogenesis in mice, we extended our study to include human seminal plasma samples. We analyzed metabolic differences between healthy males and individuals diagnosed with azoospermia. Azoospermia is a common cause of male infertility and is characterized by impaired spermatogenesis and a significant reduction in sperm count. Metabolomic approaches can provide a comprehensive view of the metabolic characteristics associated with reproductive dysfunction, potentially unveiling insights into its pathological mechanisms [29,30]. We collected seminal plasma samples from 109 healthy males and 80 azoospermia patients to investigate the metabolic differences and their underlying biological mechanisms. The data for the recruited individuals are summarized in Supplementary Table S6. We evaluated the clinical testicular biopsy samples from all azoospermia patients using H&E staining to determine the stage of spermatogenic arrest (Figure 5A–D). Among the 80 azoospermia patients included in the metabolomic analysis, 18 were diagnosed with OA (obstructive azoospermia), 39 with SCO (Sertoli-cell-only syndrome), 11 with MA (maturation arrest), and 12 with HS (hypospermatogenesis).

**Figure 6:**
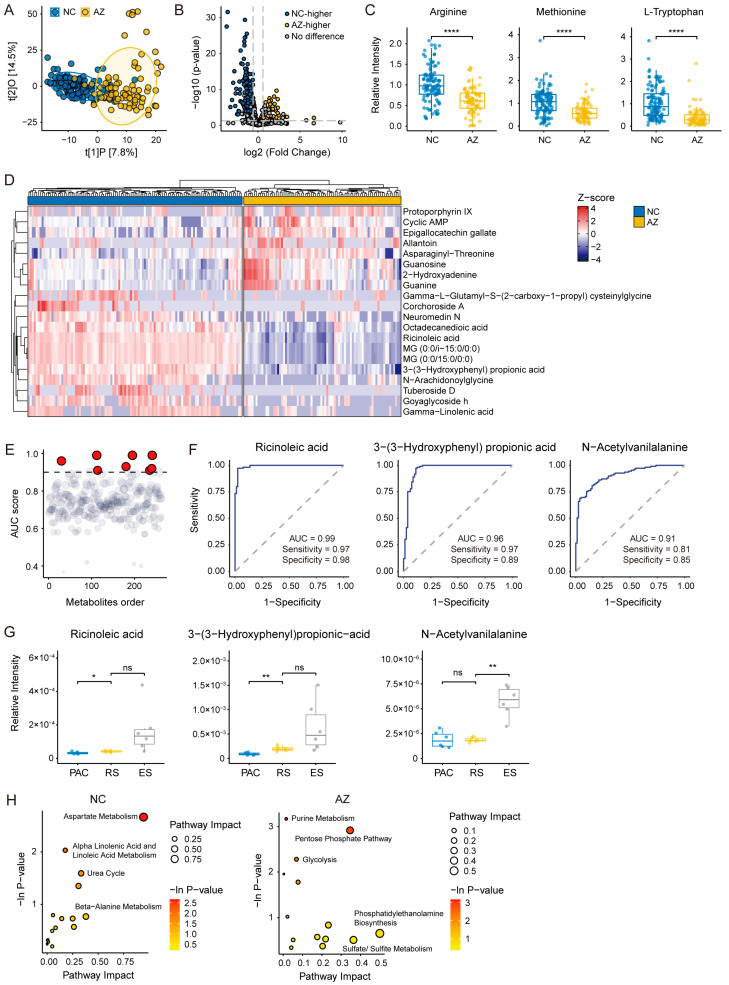
Metabolomic profiling of human seminal plasma. (**A**) OPLS-DA score plot showing clear metabolic separation between the NC (blue) and AZ (yellow) groups (R^2^X = 0.223, R^2^Y = 0.679, Q^2^ = 0.588). NC, normal control; AZ, azoospermia. (**B**) Volcano plot illustrating the differential metabolites between NC and AZ. Blue dots represent metabolites with higher abundance in the NC group, while yellow dots represent metabolites elevated in the AZ group. Dashed lines represent thresholds of log2 fold change and *p*-value. (**C**) Box plots depicting the relative intensities of three key metabolites (Arginine, Methionine, and L-Tryptophan) that are significantly lower in the AZ group compared to the NC group. (**D**) Heatmap showing the top 20 differential metabolites between the NC and AZ groups. Metabolite abundance is standardized using Z-scores. (**E**) Area under the curve (AUC) scores for all differential metabolites. The red dots represent the top-ranking metabolites with AUC > 0.9. Dashed lines represent AUC = 0.9. (**F**) Receiver operating characteristic (ROC) curves for four metabolites selected from the top-ranked candidates with the highest AUC values, including Ricinoleic acid (AUC = 0.99), 3-(3-Hydroxyphenyl) propionic acid (AUC = 0.96), N-Acetylvanilalanine (AUC = 0.91), and L-cis-Cyclo(aspartylphenylalanyl) (AUC = 0.93). The dashed line represents random classifier performance (AUC = 0.5). (**G**) Box plots showing the relative intensities of three differential metabolites, identified based on patient data, across three key spermatogenic cell types in mice (PAC, RS, and ES). Statistical significance was determined using an independent *t*-test. The boxes represent the IQR, with the horizontal line indicating the median value. Whiskers extend to 1.5 times the IQR, and individual data points are shown as jittered dots. ns, *p*-value ≥ 0.05; *, *p*-value < 0.05; **, *p*-value < 0.01; ****, *p*-value < 0.0001, respectively. (**H**) Pathway enrichment analysis of differential metabolites in the NC and AZ groups.

We analyzed the metabolic profiles of the normal control (NC) and azoospermia groups (AZ) using OPLS-DA, and the results reveal a significant separation between them (Figure 6A), suggesting substantial differences in their metabolic conditions. The volcano plot demonstrates the distribution of differential metabolites, where 203 showed increased levels in normal control subjects and 59 showed increased levels in the seminal plasma of azoospermia patients (Figure 6B). This comprehensive examination identified several metabolites that could be intricately linked to impaired spermatogenesis in azoospermia patients.

Based on the identified metabolic variations between the normal and azoospermia groups, our examinations focused on key metabolites, such as methionine, tryptophan, and arginine, which are important for spermatogenesis [31]. Further, these metabolites are significantly reduced in azoospermia patients, which underlines disruptions in the proliferation and differentiation of germ cells, as well as sperm maturation (Figure 6C). The observed reduction in arginine, for instance, may directly impair the functional maturation of sperm, as evidenced in the previously described mouse spermatogenesis process. These observations support the notion that metabolic dysregulation in azoospermia patients is fundamental to the clinically observed impaired spermatogenesis.

Furthermore, we constructed a heatmap using the 20 most differential metabolites (Figure 6D, Supplementary Table S7), illustrating the significant metabolic distinction between the normal control group and azoospermia patients. To further identify potential biomarkers, we calculated the AUC (Area Under Curve) values for each metabolite (Figure 6E). Amongst the identified potential biomarkers, ricinoleic acid, 3-(3-hydroxyphenyl) propionic acid, and N-acetylvanilalanine exhibited AUC values greater than 0.9, indicating their strong capability to distinguish the two groups (Figure 6F). To associate these results with sperm development, we examined the dynamic changes in these metabolites throughout various stages of mouse spermatogenesis (Figure 6G). The identified metabolites showed low levels during PAC, but they partially recovered in the RS and ES stages. The observed temporal pattern suggests that the reduction in these metabolites in azoospermia patients may be attributed to the absence or dysfunction of elongated spermatids, which is consistent with the clinically observed impaired spermatogenesis.

To investigate the biological significance of these metabolites, we performed a pathway enrichment analysis to elucidate their involvement in various metabolic pathways (Figure 6H, Supplementary Tables S8 and S9). The enriched pathways in the normal control group included aspartate metabolism, the urea cycle, linoleic acid metabolism, and β-alanine metabolism, highlighting the metabolic requirements during normal spermatogenesis, particularly in relation to amino acid synthesis and energy production. Conversely, the metabolic pathways that showed increased activity in the azoospermia group included purine metabolism, pentose phosphate pathway, glycolysis, and phosphatidylethanolamine biosynthesis. This enhanced activity suggests that azoospermia patients may exhibit metabolic abnormalities in the nucleic acid metabolism, carbohydrate metabolism, and membrane formation, which could adversely affect spermatogenesis.

These findings suggest that metabolic dysregulation, particularly in amino acid synthesis, energy production, and nucleic acid metabolism, is integral to impaired spermatogenesis in azoospermia patients. These metabolic abnormalities may also serve as potential biomarkers for diagnosing reproductive dysfunction.

## 3. Discussion

Here, we isolated and characterized the distinct metabolic profiles of key cell types, including PAC, RS, and ES, which are involved in mouse spermatogenesis. The metabolic profiling revealed discernible signatures across PAC, RS, and ES. Moreover, the dynamic shifts in metabolite concentrations and pathways were identified throughout the different stages of spermatogenesis, indicating a key requirement of complex metabolic orchestration for sperm maturation and functionality.

Our findings regarding metabolic changes during spermatogenesis are in good agreement with earlier reported studies and contribute to an extended understanding of certain underlying mechanisms. For example, the decrease in methylation-related metabolites during the transition from PAC to RS emphasizes the key role of DNA methylation [12,13] in regulating gene expression pertinent to spermatogenesis. This observation also corroborates recent studies underscoring the influence of epigenetic landscapes on germ cell development.

Metabolic transitions during spermatogenesis reveal stage-specific regulatory patterns that support cellular differentiation and maturation. Our findings emphasize the dynamic interplay between glycolysis, TCA cycle, and arginine metabolism, specifically during the metabolically active ES stage. The upregulation of glycolytic intermediates and enzymes, along with enhanced TCA cycle activity, emphasizes the increased energy demand, which is essential for the functional maturation of sperm. Concurrently, the elevated levels of L-arginine and related metabolites, coupled with the increased synthesis of nitric oxide, stress the critical role of nitrogen metabolism in facilitating physiological processes exclusively associated with spermatogenesis. The distinct metabolic profile at the PAC stage was distinguished by proline synthesis and metabolism. We integrated metabolomic and proteomic data to obtain a comprehensive map of metabolic regulation during spermatogenesis.

The analysis of somatic cells, particularly Leydig and Sertoli cells, further reinforces the metabolic specialization within testicular environments. The higher level of steroid hormone precursors in Leydig cells corroborates their established functional role in testosterone production, which is pivotal for spermatogenesis. In contrast, the metabolic profiles of Sertoli cells imply their integral role in sustaining germ cell development through metabolic provisioning. These distinctions are supported by previous reports highlighting the interdependence of somatic and germ cells during spermatogenesis [21,22,23]. However, it is worth noting that Leydig and Sertoli cells underwent short-term in vitro culture as part of the isolation protocol. While this brief culture period likely minimizes substantial alterations to their native metabolic states, subtle effects on their metabolic profiles cannot be entirely ruled out and should be considered in the interpretation of the results.

Finally, extending our analysis to human semen samples has important implications for understanding male infertility, particularly in cases of azoospermia. These findings demonstrate considerable metabolic divergence between normal and azoospermic samples, suggesting that metabolic disturbances could be used as biomarkers to diagnose reproductive disorders. Furthermore, the connection between altered metabolic profiles and reproductive health indicates the potential of metabolomics as a diagnostic tool in reproductive medicine.

Our findings advance the understanding of metabolic regulation during spermatogenesis by establishing a stage-specific metabolomic profile for PACs, RSs, and ESs in mice. The integration of untargeted metabolomics with cell-type-specific markers facilitates the mapping of the metabolic dependencies unique to each stage and identifies potential biomarkers for assessing germ cell development. The in-depth metabolic mapping performed in this study has immediate implications for improving fertility treatments, offering potential avenues for therapeutic intervention by targeting specific pathways at defined spermatogenic stages. Furthermore, our findings contribute to a broader field of reproductive biology by elucidating the metabolic shifts underlying the spermatogenesis process. Overall, a foundational framework can be used to enhance the diagnosis and treatment of metabolic dysfunctions associated with male infertility.

## 4. Methods and Materials

### 4.1. Ethical Statement and Study Approval

This study fully complies with government policies and the Declaration of Helsinki, and the research procedures were approved by the Ethics Committee of Jiangsu Province Hospital (Ethics No. 2023-SZ-01) on [27 June 2023]. The human semen plasma and testicular biopsy samples were obtained from participants from the Reproductive Medicine Center of Jiangsu Province Hospital. Each participant fully understands the purpose of this study and has received written consent from each participant. All participants are of Han nationality. Questionnaires were used to collect the information of participants, including personal background, lifestyle, occupational and environmental exposures, genetic risk factors, reproductive status, and medical history.

### 4.2. Mice

All animal experiments were conducted following the ethical guidelines of Nanjing Medical University. The Institutional Animal Care and Use Committee (IACUC) of Nanjing Medical University examined and approved the animal protocol (No. 2009002). The strain of mice used in the experiment was C57BL/6. Mice were housed in a specific pathogen-free (SPF) environment at the Animal Core Facility of Nanjing Medical University, with a constant temperature of 20–26 °C and relative humidity of 40–70%. Sufficient nutrients and water were provided.

### 4.3. Purification of Mouse Spermatocytes and Spermatids

Germ cells were isolated from adult male mice using the STAPUT method [10]. Cell purity of all cell types was determined by morphological evaluation and confirmed using fluorescence staining with specific markers. To determine the purity of the cells obtained using the STAPUT method, the isolated PAC, RS, and ES were applied onto glass slides and air-dried. The nuclei were then stained with Hoechst 33342 (H3570, Invitrogen, Waltham, MA, USA) for 10 min at a 1:1000 dilution. Nuclear morphology was then observed under a microscope, and the percentage of PAC, RS, and ES relative to the total cell population was calculated for each group. The graphs were plotted using GraphPad Prism (Version 9.3.1).

### 4.4. Primary Culture of Mouse Testicular Somatic Cells

To obtain Leydig cells, we used an improved protocol for isolation of primary Leydig cells from adult male mouse testicular tissues by dissociation with collagenase IV (17104019, Gibco, Waltham, MA, USA) [32]. After that, Leydig cells were cultured in vitro using Dulbecco’s Modified Eagle Medium (DMEM) (11995065, Gibco) supplied with 10% Fetal Bovine Serum (FBS) (10099141, Gibco) for at least 48 h. For further experiments, we used TrypLE™ Select (12563011, Gibco) to dissociate adherent Leydig cells into a single-cell suspension.

To obtain Sertoli cells, we used a two-step enzymatic dissociation protocol for the isolation of primary Sertoli cells from 7-day-old mouse testicular tissues [33]. After that, Sertoli cells were cultured in vitro using Dulbecco’s Modified Eagle Medium/Nutrient Mixture F-12 (DMEM/F-12) (11330032, Gibco) with 10% FBS. For further experiments, we used TrypLE™ Select to dissociate adherent Sertoli cells into a single-cell suspension.

### 4.5. Immunofluorescence and Imaging

Cell suspensions were dropped onto slides, dried, and fixed with 4% (*w*/*v*) paraformaldehyde (PFA) solution for 30 min at room temperature. The slides were then washed using PBS three times, blocked with 5% BSA, and incubated with primary antibodies at 4 °C overnight. The primary antibodies used included anti-γ-H2AX (ab26350, Abcam, Cambridge, UK), anti-3βHSD (15516-1-AP, Proteintech, Rosemont, IL, USA), anti-CYP17A1 (14447-1-AP, Proteintech), anti-VIMENTIN (AF2105, R&D SYSTEMS, Minneapolis, MN, USA), anti-SOX9 (AB5535, Millipore, Burlington, MA, USA), and anti-GATA4 (sc-25310, Santa Cruz, Dallas, TX, USA). The sections were washed with PBS and then incubated with secondary antibodies for 2–3 h at room temperature. The secondary antibodies used included IgG Alexa Fluor 488 (A-21202, Invitrogen), IgG Alexa Fluor 555 (A-31572, Invitrogen), and IgG Alexa Fluor 647 (A-21447, Invitrogen). The nuclei were stained using Hoechst 33342 for 10 min at a 1:1000 dilution. The sperm acrosomes were stained using Peanut Agglutinin (PNA) (RL-1072, Vector laboratories, Newark, CA, USA) for 60 min at a 1:500 dilution. The photographs were visualized using the Zeiss LSM800 laser-scanning microscope(Carl Zeiss, Oberkochen, Baden-Württemberg, Germany).

### 4.6. Purity of Somatic Cells

The purity of Sertoli cells was calculated as the ratio of VIMENTIN^+^ SOX9^+^ GATA4^+^ cells to the total number of cells. The purity of Leydig cells was calculated as the ratio of 3βHSD ^+^ cells to the total number of cells. The graphs were plotted using GraphPad Prism (Version 9.3.1).

### 4.7. Histological Analysis

The clinical testicular biopsy samples were fixed in modified Davidson’s fluid (MDF) solution for 48 h at room temperature, washed in 70%, 80%, 90%, and 100% ethanol sequentially, and then washed in xylene: ethanol 1:1 and 100% xylene. The fixed samples were embedded in paraffin and were cut into 10 μm slices for histology. Hematoxylin-eosin (HE) staining was performed using a hematoxylin staining solution (G1005, Service-bio, Wuhan, China) and eosin staining solution (E607321, Sangon Biotech, Shanghai, China).

### 4.8. Semen Plasma Collection

In total, 80 azoospermia patients from the Reproductive Medicine Center of Jiangsu Province Hospital were selected, with an average age of 30 (range: 23–44 years of age). The diagnosis of azoospermia was based on the 2010 World Health Organization (WHO) semen analysis guidelines [34], which define azoospermia as the absence of sperm in the pellet after at least three centrifugations at 3000 rpm for 15 min. Exclusion criteria included the following: (1) patients with reproductive system malformations, trauma, tumors, or infections; (2) patients with chromosomal abnormalities or Y chromosome microdeletions; and (3) patients with metabolic disorders such as diabetes, liver disease, or those exposed to occupational medications and other known factors related to male infertility and metabolism. Any case meeting these criteria was excluded.

At the same time, 109 semen samples from healthy control subjects undergoing routine physical examinations were selected, with semen parameters falling within the normal ranges of WHO guidelines. Control subjects were, on average, age 30 (range: 23–42 years of age). Semen parameters for control subjects met the following criteria: pH 7.2–8.0, volume ≥ 1.5 mL, sperm concentration ≥ 15 million/mL, progressive motility ≥ 32%, and normal morphology ≥ 4%.

Semen samples were collected via masturbation following 2–7 days of abstinence, using sterile, wide-mouthed, and metal-free glass containers. After collection, the semen was liquefied at 37 °C for 30 min, followed by routine analysis using a computer-assisted semen analyzer (CASA). Parameters measured included volume, sperm concentration, total sperm count, sperm motility, and progressive motility.

### 4.9. Semen Plasma Purification

Semen was liquefied at 37 °C for 30 min, followed by centrifugion at 3000 rpm for 15 min at 4 °C. The supernatant was collected and frozen at −80 °C for future use. After thawing, the semen supernatant was centrifuged at 1600× *g* for 5 min, followed by a second centrifugation at 16,000× *g* for 5 min at 4 °C. The final supernatant (seminal plasma) was stored at 4 °C for subsequent experiments.

### 4.10. Metabolomics Analysis

Samples were treated by mixing water and methanol in a 1:4 volume ratio using a homogenizer. The mixture was vortexed for 30 s to precipitate proteins, and the samples were centrifuged at 16,000× *g* for 15 min at 4 °C. The supernatant was collected and dried using a centrifugal evaporator (Labconco, Kansas City, MO, USA). Dried samples were reconstituted according to analysis requirements and prepared for UPLC.

UPLC-MS analysis was performed using a Hypersil GOLD C18 column (100 mm × 2.1 mm, particle size 1.9 μm, Thermo Fisher Scientific, Waltham, MA, USA). The mobile phases consisted of acetonitrile (0.1% formic acid) as phase A and ultra-pure water (0.1% formic acid) as phase B, with a flow rate of 0.40 mL/min and gradient elution. Ionization was achieved using a heated electrospray ionization (HESI) source with a spray voltage of 3.5 kV for positive ion mode and 2.5 kV for negative ion mode. The capillary temperature was set to 250 °C, heater temperature to 25 °C, sheath gas flow to 50 AU, auxiliary gas flow to 13 AU, and sweep gas flow to 0 AU. The S-lens voltage was set to 60 V. Full-scan mode was used with a scan range from 70 to 1050 m/z and a resolution of 70,000.

Metabolite identification was performed using TraceFinder software (v3.1, Thermo Fisher Scientific) by comparing accurate molecular weights and retention times of detected metabolites with standards.

### 4.11. Statistics and Reproducibility

Data analysis was primarily conducted using R. Principal component analysis (PCA), and orthogonal partial least squares discriminant analysis (OPLS-DA) was performed using the ropls package (v1.28.2). The identification of differentially expressed metabolites was performed using an independent two-sample *t*-test implemented in the stats package (v4.2.0). Metabolites were considered to show statistically significant changes when the *p*-value was less than 0.05. Heatmaps were generated using the ComplexHeatmap package (v2.15.3) [35]. Enrichment analysis of differential metabolites was conducted using MetaboAnalystR (v4.0.0) [36]. The pROC package (v1.18.0) was used for ROC curve analysis and AUC calculation.

## Data Availability

The original contributions presented in this study are included in the article/Supplementary Material. Further inquiries can be directed to the corresponding authors.

## Supplementary Materials

The following supporting information can be downloaded at: https://www.mdpi.com/article/10.3390/ijms26031001/s1.
